# *In vitro* effect of low-fluoride toothpaste supplemented with sodium trimetaphosphate, xylitol, and erythritol on enamel demineralization

**DOI:** 10.1590/1678-7757-2022-0410

**Published:** 2023-03-31

**Authors:** Letícia Gonçalves Oliveira, Alberto Carlos Botazzo Delbem, Francyenne Maira Castro Gonçalves, Gabriela Leal Peres Fernandes, Mark L. Cannon, Marcelle Danelon

**Affiliations:** 1 Universidade Estadual Paulista Faculdade de Odontologia Departamento de Odontologia Restauradora e Preventiva Araçatuba SP Brasil Universidade Estadual Paulista (UNESP), Faculdade de Odontologia, Departamento de Odontologia Restauradora e Preventiva, Araçatuba, SP, Brasil.; 2 Northwestern University Feinberg School of Medicine Ann and Robert Lurie Children's Hospital Chicago IL USA Northwestern University, Feinberg School of Medicine, Ann and Robert Lurie Children's Hospital, Chicago, IL, USA.; 3 Universidade de Ribeirão Preto Faculdade de Odontologia Ribeirão Preto SP Brasil Universidade de Ribeirão Preto (UNAERP), Faculdade de Odontologia, Ribeirão Preto, SP, Brasil.

**Keywords:** Dental enamel, Fluoride, Phosphate, Xylitol, Erythritol, Tooth demineralization

## Abstract

Regular use of toothpaste with fluoride (F) concentrations of ≥ 1000 ppm has been shown to contribute to reducing caries increment. However, when used by children during the period of dental development, it can lead to dental fluorosis.

**Objective:**

In this study, we aimed to evaluate the *in vitro* effect of a toothpaste formulation with reduced fluoride (F) concentration (200 ppm) supplemented with sodium trimetaphosphate (TMP: 0.2%), Xylitol (X:16%), and Erythritol (E: 4%) on dental enamel demineralization.

**Methodology:**

Bovine enamel blocks were selected according to initial surface hardness (SHi) and then divided into seven experimental toothpaste groups (n=12). These groups included 1) no F-TMP-X-E (Placebo); 2) 16% Xylitol and 4% Erythritol (X-E); 3) 16% Xylitol, 4% Erythritol and 0.2%TMP (X-E-TMP); 4) 200 ppm F (no X-E-TMP: (200F)); 5) 200 ppm F and 0.2% TMP (200F-TMP); 200 ppm F, 16% Xylitol, 4% Erythritol, and 0.2% TMP (200F-X-E-TMP); and 7) 1,100 ppm F (1100F). Blocks were individually treated 2×/day with slurries of toothpastes and subjected to a pH cycling regimen for five days (DES: 6 hours and RE: 18 hours). Then, the percentage of surface hardness loss (%SH), integrated loss of subsurface hardness (ΔKHN), fluoride (F), calcium (Ca), and phosphorus (P) in enamel were determined. The data were analyzed by ANOVA (1-criterion) and the Student-Newman-Keuls test (p<0.001).

**Results:**

We found that the 200F-X-E-TMP treatment reduced %SH by 43% compared to the 1100F treatments (p<0.001). The ΔKHN was ~ 65% higher with 200F-X-E-TMP compared to 1100F (p<0.001). The highest concentration of F in enamel was observed on the 1100F treatment (p<0.001). The 200F-X-E-TMP treatment promote higher increase of Ca and P concentration in the enamel (p<0.001).

**Conclusion:**

The association of 200F-X-E-TMP led to a significant increase of the protective effect on enamel demineralization compared to the 1100F toothpaste.

## Introduction

Dental caries remains the main problem in Dentistry due to its high prevalence in the population,^1^ and it has been considered the most common oral disease in childhood.^2^ Regular use of toothpaste with fluoride (F) concentrations of ≥1000 ppm contributes to reducing caries increment.^3^ However, when used by children during the period of dental development, it can lead to the appearance of dental fluorosis. Beyond the recommendations of supervised brushing, the use of small amounts of the toothpaste, and small orifices in the packaging,^4,5^ the reduction of fluoride concentration in toothpaste is an alternative to avoid chronic toxicity. However, that implies association with other agents to produce comparable anticariogenic effectiveness to standard market toothpaste (1100 ppm F).

Previous studies have shown that it is possible to increase the efficacy of formulations with half and even ¼ of the F concentration of toothpastes with 1100 ppm F via the addition of cyclic phosphates, such as sodium trimetaphosphate (TMP).^6,7^ However, even adding TMP to 250 ppm F toothpaste was found to show a similar effect as 1100 ppm F in reducing demineralization of tooth enamel.^7,8^ For clinical results,^6^ it is necessary for these formulations to present superior results compared to 1100 ppm F in both *in vitro* and *in situ* studies.^9^,^10^,^11^ A recent *in situ* study showed that 200 ppm F combined with TMP, xylitol (X), and erythritol (E)^12^, in a formulation called 200F-TMP-XE, resulted in a ~ 59% increase in the Ca2+ concentration in enamel compared to 1100F toothpaste. The 200F-TMP-XE and XE treatments also reduced extracellular polysaccharide production by 39% and 58%, respectively, compared to the 1100F and 200F groups. The use of polyols in toothpaste formulations to reduce the production of extracellular polysaccharides and to retain calcium in the biofilm was essential to improve the effects of low-fluoride toothpaste. However, this increase in effectiveness was not reflected in increased calcium and phosphate in enamel or a reduction in mineral loss.

Therefore, understanding the contribution of polyols in reducing enamel demineralization in the absence of biofilm utilizing low-fluoride toothpaste containing TMP will help improve new formulations. Thus, this *in vitro* study aimed to evaluate the effect of 200 ppm F, TMP, xylitol, and erythritol toothpaste on dental enamel demineralization. The null hypothesis was that the toothpaste containing F, TMP, xylitol, and erythritol would not result in additional reduction in enamel demineralization compared to conventional toothpaste containing 1100 ppm F.

## Methodology

### Experimental design

**Figure 1:**
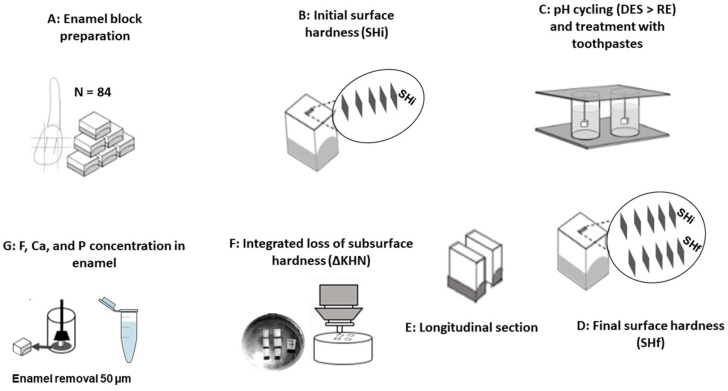
A - Enamel block preparation. B - Initial surface hardness (SHi). C - pH cycling (DES>RE) and treatment with toothpastes. D - Final surface hardness (SHf). E - Longitudinal section. F - Integrated loss of subsurface hardness (∆KHN). G - F, Ca, and P concentration in enamel

Enamel blocks (4×4×2 mm, n=84) (Figure 1A) obtained from bovine incisors were stored in 2% formaldehyde solution with a pH of 7.0 for 30 days at room temperature.^13^ The blocks were flattened and polished in a grinder polisher (BETA polisher, Buehler, Lake Bluff, Illinois, USA) with silicon carbide sandpaper in 600, 800, and 1200 grains (Extec Corp, Enfield, CT, USA), under constant water irrigation. Final polishing was carried out with felt disks moistened with 1 μm diamond solution (Extec Corp. Enfield, CT, USA). At the end of each sandpaper use, the blocks were cleaned with deionized water using ultrasound (Unique USC 1400, Indaiatuba, SP, Brazil), operated at 40 Hz and 135 W for 20 min at room temperature;^14,15^ then, the blocks were selected by initial surface hardness (SHi) (Figure 1B) test and randomly distributed (360 to 380 KHN; p=0.275) into seven groups (n=12), according to the following toothpastes: 1) no F-TMP- X-E (Placebo); 2) 16% Xylitol and 4% Erythritol (X-E); 3) 16% Xylitol, 4% Erythritol and 0.2%TMP (X-E-TMP); 4) 200 ppm F (no X-E-TMP: (200F)); 5) 200 ppm F and 0.2% TMP (200F-TMP); 6) 200 ppm F, 16% Xylitol, 4% Erythritol and 0.2% TMP (200F-X- E-TMP); and 7) 1,100 ppm F (1100F). Blocks were subjected to a pH cycling regimen (DES/RE) and treatment with toothpastes slurries 2×/day for five consecutive days and, then, they were immersed in a fresh remineralizing solution for two additional days,^7^ totalizing seven days (Figure 1C). After pH cycling, final surface hardness (SHf) (Figure 1D), percentage of surface hardness loss (%SH), integrated loss of subsurface hardness (ΔKHN) (Figure 1E-F), fluoride (F), calcium (Ca), and phosphorus (P) (Figure 1F) in enamel were determined.

### Formulation, pH determination, and F concentration in experimental toothpastes

The experimental toothpastes in the study were prepared by Xlear Incorporation, American Fork, UT, USA; they contain xylitol (X), erythritol (E), sodium trimetaphosphate (TMP), and sodium fluoride at a concentration of 200 ppm F and 1100 ppm F. A toothpaste with no added F/X/E/TMP (Placebo) was prepared using the same formulation as the others. The amounts of total fluoride (TF) and ionic fluoride (IF) were determined^11^ using a F- specific electrode (Orion 9609-BN; Orion Research Inc., Beverly, Mass., USA) connected to an ion analyzer (Orion 720 A+; Orion Research Inc.). The pH levels of toothpaste slurries were determined using a pH electrode (2A09E, Analyser, São Paulo, Brazil) calibrated with standard pH levels of 7.0 and 4.0.^16^

### pH cycling (DES>RE) and treatment with toothpastes

Before pH cycling, two layers of acid-resistant nail varnish (Risqué®-Brazil) were applied to the sides (cut surfaces) and bottom of each enamel block. Then, the blocks were individually placed in vials and subjected to five pH cycles for five consecutive days (one cycle/ day), under static conditions at 37°C. After each cycle, the blocks were immersed in a fresh remineralizing solution for two additional days,^7^ totalizing seven days of treatment. Afterwards, the blocks were immersed under constant stirring in suspensions of 1g toothpaste and 3 mL deionized water (1:3-weight:weight, for 1 min), when removed from the demineralizing (6 hours – Ca and P 2.0 mmol L^−1^ in acetate buffer 0.075 mol L^−1^, 0.04 μg F/mL in pH 4,7 – 2.2 ml/mm^2^) and from the remineralizing solutions (18 hours – Ca 1.5 mmol L^−1^, P 0.9 mmol L^−1^, KCl 0.15 mol L^−1^ in cacodylic buffer 0.02 mol L^−1^, 0. 05 μg F/mL in pH 7.0 – 1.1 m L/mm^2^).^17^ The blocks were washed with jets of deionized water for 30 seconds and dried with absorbent paper each time they were removed from the pH cycling solutions or toothpaste suspensions. After treatment, the blocks were stored in a moistened environment with deionized water at 4°C for later analysis of surface hardness.

### Hardness Analysis

Surface hardness was determined before (SHi) and after (SHf) pH cycling using a Micromet 5114 hardness tester (Buehler, Lake Bluff, USA and Mitutoyo Corporation, Kanagawa, Japan) and the Buehler OmniMet software (Buehler, Lake Bluff, USA) with a Knoop diamond indenter under a 25-g load for ten seconds. Before pH cycling, five indentations spaced 100 μm apart were produced in the center of enamel blocks to determine initial surface hardness (SHi). After pH cycling, five new indentations were produced, spaced 100 μm from the baseline indentations, to determine the final surface hardness (SHf). The percentage of surface hardness loss (%SH) was calculated using the formula %SH = [(SHf-SHi)/ SHi]×100.

For cross-sectional hardness measurements, the enamel blocks were longitudinally sectioned through their center, embedded in acrylic resin, and gradually polished. One sequence of 14 indentations, at different distances (5, 10, 15, 20, 25, 30, 40, 50, 70, 90, 110, 130, 220, and 330 μm), was made from the surface of the enamel in the central region spaced 100 μm from each other under a 25-g load for ten seconds.^7^ The Integrated hardness (KHN × μm) for the lesion into sound enamel was calculated by the trapezoidal rule (GraphPad Prism, version 3.02) and subtracted from the integrated hardness for sound enamel to obtain the integrated area of the subsurface regions in the enamel, obtaining the integrated loss of subsurface hardness (∆KHN; KHN × μm).^13^

### Analysis of the F, Ca, and P concentration in enamel

One of the halves of the longitudinally sectioned enamel blocks was sectioned again to obtain blocks with a thickness of 2 mm × 2 mm, which were fixed with adhesive glue in a mandrel for a straight piece. A layer of enamel (~ 50 µm) was then removed by microabrasion using a modified microscope base with an attached digital manometer (Pantec, São Paulo, Brazil) and 400 grit self-adhesive sanding disc (13 mm diameter) made of silicon-carbide(Buelher) fixed in crystal polystyrene flasks (J-10, Injeplast, Brazil).^16^ The resulting enamel powder retained on the polishing discs was then agitated for 1 h after adding 0.5 mL of 0.5 mol L^−1^ HCl. For F analysis, a specific electrode 9409BN (Thermo Scientific, Beverly, USA) and microelectrode reference (Analyser, São Paulo, Brazil), coupled to an ion analyzer (Orion 720A+, Thermo Scientific, Beverly, USA), were used. The electrodes were calibrated with standards ranging from 0.25 to 4.00 ppm of F (100 ppm F, Orion 940,907) under the same conditions as the samples. The readings were conducted in 0.300 mL of the biopsy solution with the same volume of TISAB II modified NaOH.^13^ The data obtained in mV were converted to μg/mm^3^. For Ca, the Arsenazo III colorimetric method was used^18^ and absorbance readings were recorded at 650 nm with a plate reader (PowerWave 340, Biotek, Winooski, USA). P was measured using the Fiske and Subbarow^19^ (1925) method, and absorbance readings were recorded at 660 nm. The results of these assays were expressed in μg/mm^3^.

### Statistical analysis

The SigmaPlot 12.0 software was used for statistical analysis with the significance level set at 5 %. All variables exhibited a normal (Shapiro–Wilk) and homogeneous (Bartlet) distribution. Data from the enamel analysis (%SH, ∆KHN, F, Ca, and P) were analyzed using a one-way analysis of variance (ANOVA). The Student–Newman–Keuls post hoc test was performed for multiple comparisons.

## Results

The mean (SD) concentrations of ionic fluoride (IF) and total fluoride (TF) (n=3) were, respectively: 1) Placebo – 10.3 (0.4) and 10.7 (1.1); 2) X-E – 15.2 (16.8) and 16.4 (40.1); 3) X-E-TMP – 13.6 (12.5) and 17.6 (20.10); 4) 200F – 200.1 (11.4) and 206.8 (10.6); 5) 200F-TMP – 208.4 (10.40) and 220.1 (11.4); 6) 200F-TMP-X-E – 204.0 (16.8) and 210.3 (18.4); and 7) 1100F – 1127.2 (15.2) and 1182.0 (28.2). The variation in fluoride concentration in the formulations was at a limit of 10% based on what had been proposed for toothpaste. The pH of the groups was 7.1 (0.1), ranging from 7.1 to 7.3.

The mean initial surface hardness (SHi) for all enamel blocks was 371.0 (1.2), varying from 361.2 (6.2) and 377.2 (5.2) KHN in the experimental groups (p=0.054). No significant differences were found among the groups after allocation (p=0.738). Placebo and X-E groups showed similar and higher %SH values (p>0.001). The 200F-X-E-TMP group reduced enamel demineralization (%SH) by 39% and 43% compared to 200F-TMP and 1100F treatments, respectively (p<0.001) (Table 1). No statistical difference was observed for the 200F-TMP and 1100F treatments (p>0.001). Lesion body reduction was similar between Placebo and X-E (p>0.001) and between 200F-TMP and 1100F (p>0.001) treatments (Table 1). The lower depth of the lesion (ΔKHN) was observed for the 200F-X-E-TMP group compared to the other groups (p<0.001), with 65% lower ΔKHN than the 1100F group (Table 1).

**Table 1:** Mean (SD) of the variables analyzed in the enamel according to the toothpastes

Toothpastes	%SH	∆KHN	F	Ca	P
	(KHN)	(KHN x µm)	(µg/mm³)	(µg/mm³)	(µg/mm³)
Placebo	-80.2^a^	8,622.4^a^	1.1^a^	203.2^a^	103.4^a^
	(4.2)	(421.6)	(0.2)	(31.6)	(12.2)
X-E	- 78.6^a^	7,999.4^a^	1.2^a^	261.4^b^	159.1^b^
	(2.2)	(437.9)	(0.1)	(18.2)	(33.8)
X-E-TMP	- 56.8^b^	5,622.5^b^	1.8^a^	254.4^b^	160.1^b^
	(1.4)	(621.7)	(0.4)	(12.4)	(36.8)
200F	- 41.6^c^	4,617.6^c^	1.7^a^	261.1^b^	160.2^b^
	(1.2)	(721.4)	(0.3)	(22.4)	(28.4)
200F-TMP	- 30.1^d^	3,027.4^d^	2.5^b^	346.7^c^	162.4^b^
	(2.1)	(544.6)	(1.3)	(32.1)	(44.1)
200F-X-E-TMP	- 18.2^e^	1,122.7^e^	2.7^b^	554.9^d^	239.1^c^
	(1.3)	(12.4)	(1.4)	(94.2)	(39.4)
1100F	- 32.1^d^	3,226.8^d^	3.5^c^	344.7^c^	163.1^b^
	(1.6)	(612.1)	(0.7)	(42.1)	(31.4)

* Distinct superscript letters indicate statistical significance among the treatment in each analysis (One-way ANOVA, followed Student–Newman–Keuls’ test). Values represent means (standard deviations). %SH: percentage of surface hardness loss. ΔKHN: integrated subsurface hardness loss. F: fluoride in the enamel. Ca: calcium in the enamel. P: phosphorus in the enamel.

Treatment with 1100F led to the highest concentration of F in the enamel (p<0.001), followed by 200F-TMP and 200F-X-E-TMP; both showed similar concentrations (p>0.001) (Table 1). Treatment with 200F-X-E-TMP promoted an increase in the concentration of Ca in the enamel by ⁓ 60% and ⁓ 61% compared to the 200F-TMP and 1100F groups, respectively (p<0.001). No significant differences were observed between the 200F-TMP and 1100F groups (p>0.001) and between X-E, X-E-TMP and 200F groups (p>0.001) (Table 1). Similar values of P in enamel were observed in X-E, X-E-TMP, 200F, 200F-TMP, 1100F groups (p>0.001); the 200F-X-E-TMP group showed the highest concentration (p<0.001).

## Discussion

Fluoride toothpastes can be used as a vehicle to improve the oral health of individuals.^3^ This study aimed to evaluate whether the addition of X-E-TMP to a 200 ppm F toothpaste would enhance its protective effect on enamel demineralization. Toothpastes containing higher concentrations of F may provide greater protection against caries but increase the risk of fluorosis. Chronic ingestion of F from toothpaste in young children is common and, despite wide variation in the amount ingested, the younger the children are, the more likely they tend to swallow larger amounts, which often represents a part of the total daily intake of fluoride and may be sufficient to cause fluorosis.^3^ However, the satisfactory results obtained from the incorporation of polyols and phosphate to a low-fluoride toothpaste could represent an effective strategy to minimize the occurrence of dental fluorosis and to increase the remineralizing capacity of toothpaste formulation since these products promote a large precipitation of calcium phosphate on the surface of carious lesions. The results showed that the addition of 16%X, 4%E, and 0.2%TMP to 200F led to superior anticaries effects when compared to the conventional toothpaste. Thus, the null hypothesis was rejected.

Xylitol and erythritol have been used as anticaries agents by depressing acidogenicity and volume in biofilm.^20^ A recent study confirmed these results since toothpaste containing 16% xylitol and 4% erythritol has been shown to reduce the amount of extracellular polysaccharides in biofilm more effectively than toothpaste with 1100 ppm F.^12^ Furthermore, the mineral loss between the two toothpastes did not differ using this *in situ* caries model. However, in abiotic caries models, the results are conflicting since 20% xylitol can induce remineralization into a deeper layer of artificial caries lesions,^21,22^ whereas erythritol does not present any effect.^23^ In the present study, the combination of 16% xylitol and 4% erythritol did not show any effect on the surface or subsurface artificial enamel demineralization lesions (Table 1), despite enamel having higher calcium and phosphate amounts than the Placebo group. Although previous studies suggest that xylitol can form complexes with calcium on enamel surface, facilitating its movement into lesion pores,^21,22^ it does not reduce acid penetration into the enamel's inner layers. Additionally, Its poor ability retention in enamel,^23^ especially in abiotic caries models, may have contributed to these results. The lower mineral loss was probably due to the ability of TMP to absorb enamel^24^ and reduce acid diffusion deep into the lesion when associating polyols to TMP, thus acting against the demineralization process.^7,10,11^ However, under the same conditions, the 200F group showed less mineral loss compared to X-E-TMP. Fluoride promotes the formation of a harder outer layer, but it does not prevent the acid diffusion into the deeper layers of the lesion,^7,10,11^ which explains similar calcium and phosphate values in enamel compared to the X-E and X-E-TMP groups. In this context, the concentrations of calcium and phosphate in the enamel measured after application of the toothpaste were not related to the concentration of F present in the treatments, that is, it was not dose-dependent on F. The presence of X and E contributes to a greater amount of calcium and phosphate in the 200F-TMP-XE and XE groups, which could be explained by the ability of X to complex these cations.

In our study, we found that the combination of F with TMP was effective in reducing enamel demineralization similarly to standard 1100F toothpaste^7,8^. Additionally, we found that toothpaste containing 0.25% TMP in combination with 250 ppm F reduced the inner part of the caries lesion (∆KHN) by 20% compared to 1100 ppm F toothpaste^7^. As stated previously, when considering a clinical study with a new formulation, it is important that it shows superior results to the standard formulation in *in vitro* and *in situ* studies. In our study, we found that treatment with the 200F-TMP toothpaste in combination with X-E led to less enamel demineralization, both on the surface (%SH: ~ 43%) and in depth (ΔKHN: ~ 65%), compared to the 1100 ppm F toothpaste (Table 1). By associating 0.2% of TMP to 200 ppm F, there is an increase in the presence of F and Ca in the enamel compared to the 200F group, as observed in previous studies.^7,8^ The addition of polyols did not change the amount of fluoride, but the concentration of Ca and P in enamel was 60% and 47%, respectively, higher compared to the 1100F group. Our findings suggest that F and TMP favor the retention of polyols to enamel, improving calcium and phosphate retention in enamel. The effect of agents with anti-caries activity depends on their ability to adsorb to the tooth structure. In an *in situ* enamel demineralization study, X-E alone was not able to retain calcium and phosphate, but only when combined with F and TMP.^13^ The combination F and TMP was reported to reduce acid diffusion and enhance the flux of calcium and phosphate into the deeper layers of the lesion.^7,11^

In this sense, it is believed that TMP retains positively charged ions of CaF^+^ and Ca^2+^, replacing Na+ in the cyclic structure and promoting reduction in acid diffusion. This interaction can change the selective permeability and diffusion of ions into the enamel. ^25,26,27^ In case of a drop in pH, it has been suggested that the release of these ions from TMP increases the ionic activity of neutral species (CaHPO_4_^0^ and HF^0^).^8^ In addition to their higher enamel diffusion coefficient, neutral species would not be retained by the enamel surface, but could penetrate the enamel, thereby decreasing enamel demineralization^25,27,28^ and increasing remineralization. Xylitol has been reported to complex cations^23^ and induce remineralization of deeper layers of enamel lesions by facilitating the diffusion of calcium^21^ and reducing the translocation of dissolved calcium and phosphate during demineralization. When TMP, X, E, and F were combined, a greater flow of Ca and P within the lesions in the deeper layers was observed, which can block the interprismatic pores in front of the lesion, thereby reducing the diffusion of acid to the underlying sound enamel, which corroborates with the finding in our study.^29^ Despite the possibility of competition for binding sites in enamel between polyols, F, and TMP, the results showed an additive effect, allowing the association of these agents. The agents were not tested separately due to their different properties, with the polyols acting on the extracellular polysaccharides and TMP having an opposite action, acting on mineral exchange, thus not having satisfactory results compared to the association.^12^ Furthermore these agents have been shown to have no abrasive potential on enamel due to their antimicrobial and remineralizing properties.^12^

We emphasize that the results obtained in this study, which do not simulate the complexity of an oral environment, provide initial evidence to encourage further exploration into the philosophy of multiple mechanisms together for caries prevention, especially for patients at high risk. Thus, some factors should be considered: pH cycling models do not faithfully reproduce the oral situation; the absence of biofilm in the model may have hindered the effectiveness of Xylitol and Erythritol to create a state of Ca ion supersaturation, which prevented this system from adequately reducing enamel demineralization.

Therefore, we recommend that future studies should consider the following factors: 1) the evaluation of biofilm and saliva (biochemical and microbiological); 2) the use of an *in situ* remineralization model; and the conduct of controlled and randomized clinical trials in different populations with diverse dietary and oral hygiene habits. These studies could provide more robust evidence on the efficacy of the association of Xylitol/Erythritol and TMP since TMP has no effect on the microbiota. In conclusion, our study found that the application of 200F-X-E-TMP promoted a superior effect against enamel demineralization when to the 1100F toothpaste.

## Data Availability

All data generated and analyzed during this study are included in this published article

## References

[1] Golubnitschaja O, Baban B, Boniolo G (2016). Medicine in the early twenty-first century: paradigm and anticipation - EPMA position paper 2016. EPMA J.

[2] (2018). Policy on Early Childhood Caries (ECC): classifications, consequences, and preventive strategies. Pediatr Dent.

[3] Walsh T, Worthington HV, Glenny AM, Marinho VC, Jeroncic A. (2019). Fluoride toothpastes of different concentrations for preventing dental caries. Cochrane Database Syst Rev.

[4] Hall KB, Delbem AC, Nagata ME, Hosida TY, Moraes FR, Danelon M (2016). Influence of the amount of dentifrice and fluoride concentrations on salivary fluoride levels in children. Pediatr Dent.

[5] Hamilton J (2001). New CDC report offers fluoride use tips. J Calif Dent Assoc.

[6] Freire IR, Pessan JP, Amaral JG, Martinhon CC, Cunha RF, Delbem AC (2016). Anticaries effect of low-fluoride dentifrices with phosphates in children: A randomized, controlled trial. J Dent.

[7] Missel EM, Cunha RF, Vieira AE, Cruz NV, Castilho FC, Delbem AC (2016). Sodium trimetaphosphate enhances the effect of 250 p.p.m. fluoride toothpaste against enamel demineralization in vitro. Eur J Oral Sci.

[8] Souza MD, Pessan JP, Lodi CS, Souza JA, Camargo ER, Souza FN (2017). Toothpaste with nanosized trimetaphosphate reduces enamel demineralization. JDR Clin Trans Res.

[9] Takeshita EM, Castro LP, Sassaki KT, Delbem AC (2009). In vitro evaluation of dentifrice with low fluoride content supplemented with trimetaphosphate. Caries Res.

[10] Takeshita EM, Danelon M, Castro LP, Sassaki KT, Delbem AC (2015). Effectiveness of a toothpaste with low fluoride content combined with trimetaphosphate on dental biofilm and enamel demineralization in situ. Caries Res.

[11] Takeshita EM, Danelon M, Castro LP, Cunha RF, Delbem AC (2016). Remineralizing potential of a low fluoride toothpaste with sodium trimetaphosphate: an in situ study. Caries Res.

[12] Marcato RA, Garbelini CC, Danelon M, Pessan JP, Emerenciano NG, Ishikawa AS (2021). In situ evaluation of 200 ppm fluoride toothpaste content trimetaphosphate, xylitol and erythritol on enamel demineralization and dental biofilm. J Dent.

[13] Emerenciano NG, Botazzo Delbem AC, Pessan JP, Nunes GP, Souza FN, Camargo ER (2018). In situ effect of fluoride toothpaste supplemented with nano-sized sodium trimetaphosphate on enamel demineralization prevention and biofilm composition. Arch Oral Biol.

[14] Favretto CO, Delbem AC, Moraes JC, Camargo ER, Toledo PT, Pedrini D (2018). Dentinal tubule obliteration using toothpastes containing sodium trimetaphosphate microparticles or nanoparticles. Clin Oral Investig.

[15] Favretto CO, Delbem AC, Toledo PT, Pedrini D. (2021). Hydraulic conductance of dentin after treatment with fluoride toothpaste containing sodium trimetaphosphate microparticles or nanoparticles. Clin Oral Investig.

[16] Danelon M, Garcia LG, Pessan JP, Passarinho A, Camargo ER, Delbem AC (2019). Effect of fluoride toothpaste containing nano-sized sodium hexametaphosphate on enamel remineralization: an in situ study. Caries Res.

[17] Weatherell JA, Robinson C, Strong M, Nakagaki H. (1985). Micro-sampling by abrasion. Caries Res.

[18] Vieira AE, Delbem AC, Sassaki KT, Rodrigues E, Cury JA, Cunha RF (2005). Fluoride dose response in pH-cycling models using bovine enamel. Caries Res.

[19] Vogel GL, Chow LC, Brown WE (1983). A microanalytical procedure for the determination of calcium, phosphate and fluoride in enamel biopsy samples. Caries Res.

[20] Fiske CH, Subarrow Y (1925). The colorimetric determination of phosphorus. J. Biol. Chem.

[21] Cock P (2018). Erythritol functional roles in oral-systemic health. Adv Dent Res.

[22] Miake Y, Saeki Y, Takahashi M, Yanagisawa T (2003). Remineralization effects of xylitol on demineralized enamel. J Electron Microsc (Tokyo).

[23] Cardoso CA, Castilho AR, Salomão PM, Costa EN, Magalhães AC, Buzalaf MA (2014). Effect of xylitol varnishes on remineralization of artificial enamel caries lesions in vitro. J Dent.

[24] Cock P, Mäkinen K, Honkala E, Saag M, Kennepohl E, Eapen A. (2016). Erythritol is more effective than xylitol and sorbitol in managing oral health endpoints. Int J Dent.

[25] Amaral JG, Pessan JP, Souza JA, Moraes JC, Delbem AC (2018). Cyclotriphosphate associated to fluoride increases hydroxyapatite resistance to acid attack. J Biomed Mater Res B Appl Biomater.

[26] Takeshita EM, Exterkate RA, Delbem AC, ten Cate JM (2011). Evaluation of different fluoride concentrations supplemented with trimetaphosphate on enamel de- and remineralization in vitro. Caries Res.

[27] Danelon M, Takeshita EM, Sassaki KT, Delbem AC (2013). In situ evaluation of a low fluoride concentration gel with sodium trimetaphosphate in enamel remineralization. Am J Dent.

[28] Danelon M, Takeshita EM, Peixoto LC, Sassaki KT, Delbem AC (2014). Effect of fluoride gels supplemented with sodium trimetaphosphate in reducing demineralization. Clin Oral Investig.

[29] Da Camara DM, Pessan JP, Francati TM, Souza JA, Danelon M, Delbem AC (2016). Fluoride toothpaste supplemented with sodium hexametaphosphate reduces enamel demineralization in vitro. Clin Oral Investig.

